# Impact of Patient Body Mass Index on Post-Operative Recovery from Robotic-Assisted Hysterectomy

**DOI:** 10.3390/cancers15174335

**Published:** 2023-08-30

**Authors:** Anumithra Amirthanayagam, Matthew Wood, Lucy Teece, Aemn Ismail, Ralph Leighton, Annie Jacob, Supratik Chattopadhyay, Quentin Davies, Esther L. Moss

**Affiliations:** 1 Leicester Cancer Research Centre, College of Life Sciences, University of Leicester, University Road, Leicester LE1 7RH, UK; 2 Department of Gynaecological Oncology, University Hospitals of Leicester NHS Trust, Infirmary Square, Leicester LE1 5WW, UK; 3 Department of Population Health Sciences, College of Life Sciences, University of Leicester, University Road, Leicester LE1 7RH, UK; 4 Department of Anaesthetics, University Hospitals of Leicester NHS Trust, Infirmary Square, Leicester LE1 5WW, UK

**Keywords:** endometrial cancer, obesity, body mass index, robotic surgery, quality of life, minimally invasive surgery

## Abstract

**Simple Summary:**

Robotic surgery is reported to have benefits for the surgical management of patients with a high BMI. However, there is a lack of information on patient-reported outcomes and recovery following robotic-assisted hysterectomy (RH). A study collecting information on participant characteristics, intra- and post-operative events was conducted. Telephone questionnaires at 2, 4, 6, and 12 weeks were used to collect patient-reported recovery using the QoR-40 quality-of-recovery questionnaire. Of the 53 individuals recruited, 50 underwent RH and three cases were converted to open surgery. Patient BMI had a small impact on operative time (*p* = 0.04) but not on length of stay (*p* = 0.62). Overall quality-of-life (QOL) scores were consistently high post-operatively, indicating a high quality of recovery, and were not impacted by patient BMI.

**Abstract:**

A longitudinal, descriptive, prospective, and prolective study of individuals with endometrial or cervical cancer/pre-cancer diagnoses and high BMI (over 35 kg/m^2^) undergoing RH was conducted. Of the 53 participants recruited, 3 (6%) were converted to open surgery. The 50 RH participants had median BMI 42 kg/m^2^ (range 35 to 60): the range 35–39.9 kg/m^2^ had 17 cases; the range 40–44.9 kg/m^2^ had 15 cases; 45–49.9 kg/m^2^ 8 cases; and those ≥50 kg/m^2^ comprised 10 cases. The mean RH operating time was 128.1 min (SD 25.3) and the median length of hospital stay was 2 days (range 1–14 days). Increased BMI was associated with small, but statistically significant, increases in operating time and anaesthetic time, 65 additional seconds and 37 seconds, respectively, for each unit increase in BMI. The median self-reported time for individuals who underwent RH to return to their pre-operative activity levels was 4 weeks (range 2 to >12 weeks). There was a significant improvement in pain and physical independence scores over time (*p* = 0.001 and *p* < 0.001, respectively) and no significant difference in scores for overall QOL, pain, or physical independence scores was found between the BMI groups. Patient-reported recovery and quality of life following RH is high in individuals with high BMI (over 35 kg/m^2^) and does not appear to be impacted by the severity of obesity.

## 1. Introduction

Rising body mass index (BMI) can potentially impact on patient care, most notably due to increased risk of complications following surgery [1]. Although many patients who require surgery for a gynaecological malignancy are classified as being within the obese category (BMI > 30 kg/m^2^), endometrial cancer (EC) has the greatest association [2]. The rising BMI levels within the population are proposed as being a contributing factor to the increasing incidence rate of EC [3]. Surgery is the primary treatment for EC. However, since anaesthetic and surgical risks [4], and consequentially intra- and post-operative morbidity, are reported to rise with rising patient BMI [5,6], it is important that patients are appropriately prepared and counselled pre-operatively. The introduction of minimally invasive surgery, whether laparoscopic (LS) or robotic-assisted surgery (RS), has been shown to have significantly more favourable peri-operative outcomes compared to open surgery [7] without impacting on oncological outcomes [8]. In particular, RS has been reported to have further advantages compared to LS, with greater degrees of movement, 3-D visualisation, tremor reduction, and a shorter learning curve for the surgeon [9]. A Cochrane review of 12 randomised collected trials (RCTs) of robotic-assisted (RH) and laparoscopic hysterectomy (LH) [10] reported comparable complications rates, although a meta-analysis of EC cases reported a lower conversion to laparotomy rate with RH compared to LH [11]. In particular, RH has been proposed as playing a role in the surgical management of patients with a very high BMI, where LH may be more challenging to perform and associated with greater adverse ergonomic impacts on the surgeon [12,13]. Several studies have published the intra-operative events and immediate post-operative recovery in high-BMI individuals undergoing RH, however, there is a lack of information on analgesia requirements, post-operative recovery times, and experiences. 

We undertook a longitudinal, descriptive, prospective, and prolective study to investigate the impact of patient BMI on RH intra-operative events, analgesia requirements, and post-operative recovery. 

## 2. Materials and Methods

The study was granted ethical approval by the London South-East Research Ethics Committee (17/LO/0915). Individuals with a BMI ≥ 35 kg/m^2^ selected for RH, with or without bilateral salpingo-oopherectomy, to treat cervical (stage IA1), endometrial cancer (Stage I), or pre-cancer (cervical intra-epithelial neoplasia or complex atypical hyperplasia) at the University Hospitals of Leicester NHS Trust between August 2017 and February 2019 were offered recruitment. The inclusion and exclusion criteria are outlined in the Supplementary Table S1.

Sentinel lymph node biopsy for EC was not available at the time of the study and para-aortic lymphadenectomy was not routinely performed in high-risk cases where there was no suspicion of pelvic lymph node involvement on MRI/CT scan. The procedures were performed by three consultant gynaecological oncologists (2–4 years’ RS experience), using the Da Vinci Si (Intuitive Surgical Lt, Sunnyvale, CA, USA). All participants provided written consent prior to enrolment. Pre-operatively, baseline demographics were obtained from medical notes, including age, BMI, and previous abdominal surgery. Prior to surgery, participant anthropometric measurements were obtained using the PLUs Size anthropometry self-Measurement Tool (PLUSMET) [14]. Intra-operative events and timings were recorded. Anaesthetic time included time for the positioning of invasive monitoring, for example arterial lines. Post-operatively, the participants were reviewed daily during their in-patient hospital stay to assess numeric pain rating scale and analgesia usage, and were managed according to enhanced recovery following surgery (ERAS) principles [15]. Post-operative complications were scored using the Clavien–-Dindo scoring system [16]. Post-operative telephone questionnaires were completed with participants at 2, 4, 6, and 12 weeks, in order to assess subjective post-operative recovery using the numeric pain rating scale and the validated quality-of-recovery questionnaire (QoR-40) [17], focusing on physical independence, pain, and overall Quality of Life (QOL). 

Data were analysed using Stata 17 (StataCorp. 2021. Stata Statistical Software: Release 17. College Station, TX, USA: StataCorp LLC). Descriptive statistics are presented to summarise the included participant characteristics. Intra-operative outcomes are summarised overall and by BMI categories. The estimated sample size to test for an association between BMI on operation time using univariable logistic regression (two-sided test with alternative hypothesis slope/regression coefficient = 1.1 with a standard error of 0.5), assuming the standard deviation of BMI = 2, with 80% power and 0.05 significance level, was calculated as *N* = 52. Univariable logistic regression models with BMI included as a continuous linear variable were fitted for each intra-operative outcome, regression coefficients and *p*-values are reported, and linear trends for operative and anaesthetic time are presented graphically. Correlations between characteristics and intra-operative outcomes were assessed using Spearman’s Rank correlation and reported. Quality-of-life measures over the four time points were analysed using repeated measures mixture models, with random intercepts and BMI included as a categorical variable. Missing data were managed through case-wise deletion, and the number of participants included in each analysis are reported. Statistical significance was considered where *p* ≤ 0.05.

## 3. Results

In total, 53 participants were recruited during the study period, and 50 (94%) received the planned surgical procedure. Three cases (6%) were converted to open surgery (BMI 35, 43, and 53); one of these was prior to the start of the surgical procedure due to anaesthetic issues, and two were before docking, due to extensive adhesions from previous surgery and identification of Stage IV disease on abdominal inspection.

The 50 participants who underwent RH had a median age of 63 years (range: 36 to 79 years) and median BMI of 42 kg/m^2^ (range: 35 to 60 kg/m^2^); see Table 1. More than half of the participants were classified as American Society of Anaesthesiologists (ASA) grade 3 (*N* = 29, 58%), 20 cases (40%) were grade 2, and 1 (2%) was grade 4. Over half (*N* = 27, 64%) had previously undergone abdominal surgery with a variety of laparoscopic and open procedures. Caesarean section was the most common procedure. Four participants (8%) reported two previous caesarean sections and one (2%) had undergone three, and two participants (4%) had undergone an umbilical hernia repair ± mesh. 

Two participants (4%) had a pelvic lymphadenectomy performed in addition to an RH. Their outcome measurements were excluded from the analysis of intra-operative timings due to increased operative time associated with the additional treatment. Intra-abdominal pressure during the surgical procedure had a median of 8 mmHg (range: 8 to 15 mmHg) and was significantly associated with BMI (univariable *p* < 0.001) (Table 2).

**Figure 1:**
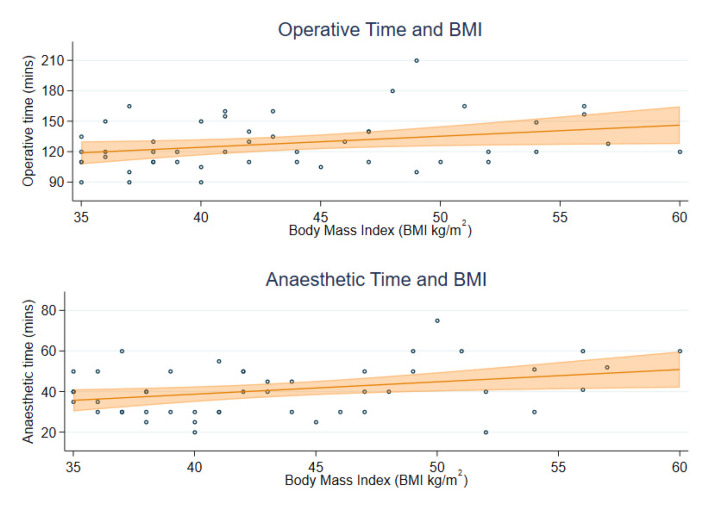
Operative and anaesthetic time by participant BMI.

For the 48 RH cases without lymphadenectomy, the mean operating time was 128.1 min (SD 25.3) and anaesthetic time was 40.8 min (SD 12.3). Increased BMI was univariably associated with small, but statistically significant, increases in operating time (65 additional seconds (95% CI: 3 to 128) for each unit increase in BMI) and anaesthetic time (37 additional seconds (95% CI: 7 to 67) for each unit increase in BMI) (Table 2). The operative and anaesthetic times for individuals with BMI of 50 kg/m^2^ are predicted to be 135.2 (95% CI: 125.4 to 145.0) and 44.8 (95% CI: 40.1 to 49.5) minutes on average, which is estimated to be 16.4 and 9.1 min longer than the average for an individual with BMI of 35 kg/m^2^ (Figure 1).

The median estimated blood loss was 100 mL (range 50 to 800 mL), and there were no intra-operative visceral injuries. In total, six participants (12%) required High Dependency Unit admission post-procedure, five (10%) electively due to known co-morbidities, and one unplanned admission due to persistent low oxygen saturations. There was only one grade 3 Clavien–Dindo complication, namely, pneumonia and fluid overload requiring non-mechanical ventilation and diuretic treatment. No other grade ≥ 3 complications were identified during the 12-week follow-up period. One participant (2%) was readmitted to the hospital two weeks post-procedure due to vaginal bleeding whilst on therapeutic anticoagulation due to a previous pulmonary embolism. Another participant required hospital admission post-operatively for an unrelated neurological condition, and four participants attended for medical review due to a vaginal bleed or urinary symptoms. 

All of the RH cases used simple analgesia (paracetamol/diclofenac/ibuprofen) on the day of surgery. Within 24 h, 27 (54%) used a weak opioid (codeine/tramadol) and 10 (20%) a strong opioid (morphine), median 10 mg morphine in total. The participants’ pain reduced over time, from a median score of 2 out of 10 on the day following surgery, to a median score of 0 after 2 weeks; this was reflected in a reduction in analgesia use. The median length of hospital stay for the RH cases was 2 nights (range 1 to 14). There was no evidence of an association between BMI and length of stay (*p* = 0.623). However, participants with an ASA classification ≥ 3 had a significantly longer stay compared to ASA 2 participants (*p* = 0.013).

The median self-reported time for RH cases to return to their pre-operative activity levels was by 4 weeks (range 2 to 12 weeks). Only one participant (2%) reported that they still had not returned to their previous activity levels at 12 weeks. Time to returning to everyday activity was not found to be associated with BMI (univariable *p* = 0.610). No significant correlations were found between time to recovery and ASA (*p* = 0.417) or age (*p* = 0.774). 

**Figure 2:**
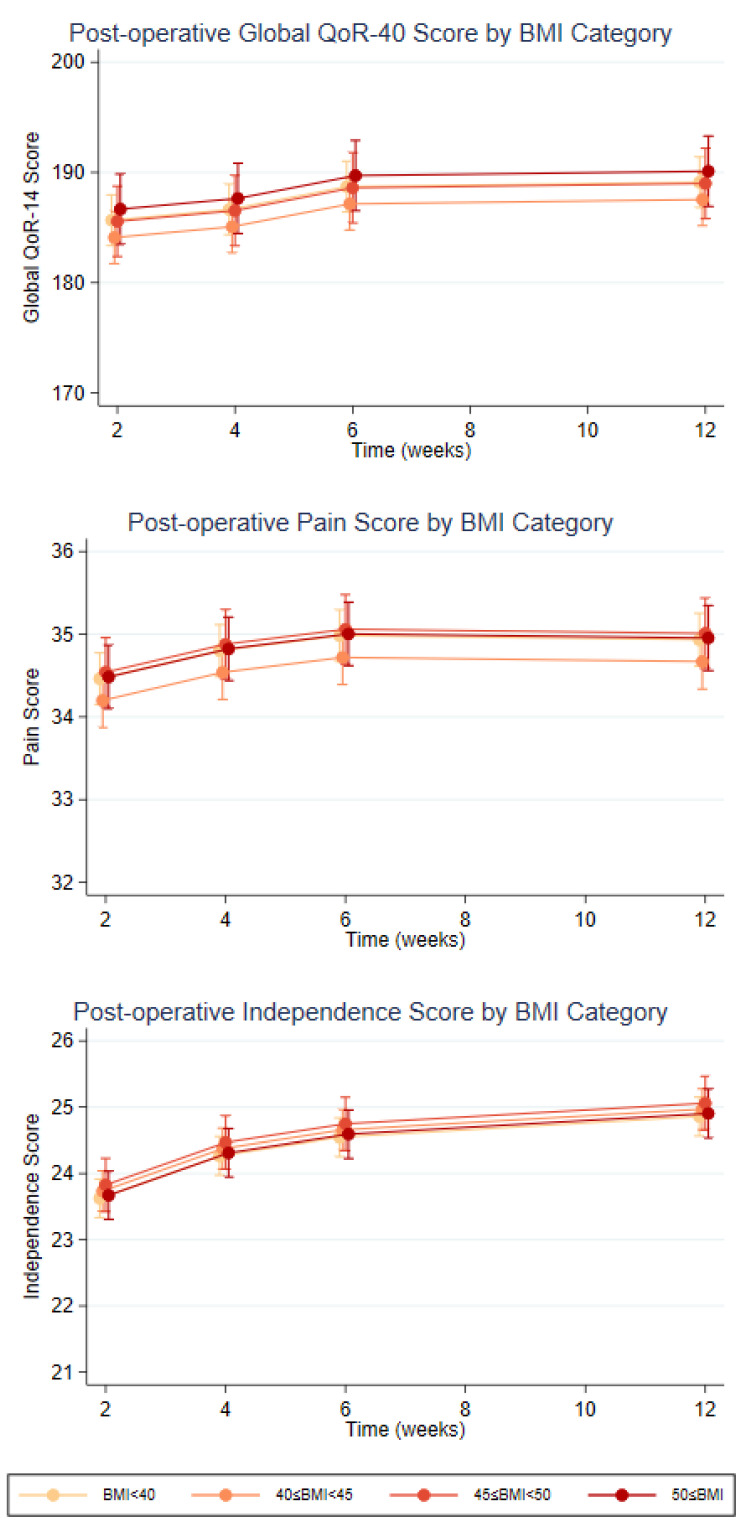
Post-Operative QOL Scores at 2, 4, 6, and 12 weeks by BMI categories.

Overall QOL scores for the participants undergoing RH were consistently high post-operatively, with a median score of 188 out of a maximum score of 200 (range 166 to 194) at 2 weeks (*N* = 49), indicating a high quality of recovery. QoR-40 data from all four time points were available for 43 participants, and this showed statistically significant improvements in total QOL scores (3.44, 95% CI 1.8 to 2.1, *p* < 0.001), pain scores (0.47, 95% CI 0.2 to 0.8, *p* = 0.002), and in physical independence score (1.23, 95% CI 1.0 to 1.4, *p* < 0.001) by 12 weeks. There was no significant difference in scores between the BMI groups from the overall QOL, pain, or physical independence scores (Figure 2).

## 4. Discussion

This pragmatic, prospective study has given an in-depth picture of intra-/post-operative events and patient-subjective recovery following RH in a population of patients with different levels of obesity. The results indicate that despite being a high-risk population there was a low risk of intra- and post-operative complications, in keeping with other case series [18], and the median subjective return to previous activity levels was only 4–6 weeks. 

The risks of surgery are reported to increase with rising patient BMI, due to increasing prevalence of medical co-morbidities [19], in particular wound infections [20], but also due to the technical challenges associated with abdominal wall depth and intra-abdominal adiposity. Minimally invasive surgery is well established as being associated with a shorter hospital stay, reduced complications and quicker patient recovery as compared to open surgery [7], including in high-BMI populations [21]. Many of the previously reported studies on the outcome of RH in patients with obesity have predominantly contained participants within the BMI 30–35 kg/m^2^ range, and therefore the impact in patients with higher BMIs can be lost in the final analysis [22]. Our study only recruited participants with a BMI ≥ 35 kg/m^2^ in order to focus on the recovery of this population. 

The majority of our study population underwent RH alone, since nodal assessment was not indicated due to pre-malignant or low-risk classification [23]. Rates of nodal assessment are reported to fall with rising BMI. For example, 95% of patients with a BMI > 50 did not have nodal assessment [24]. Sentinel lymph node mapping, however, has been reported to be successful in 85.6% of patients with a BMI 40–49.9 kg/m^2^ and 73.9% of BMI > 50 kg/m^2^ [18]. Lymphadenectomy can be more challenging with rising BMI, in particular para-aortic node assessment, and may result in patients being incompletely staged, although centralising cases to high-volume centres and increasing familiarity with sentinel node mapping is likely to increase the proportion of patients who undergo complete staging in the future [25]. 

RH is often reported to be associated with longer operative times compared to open hysterectomy or LH [26], although this is reported to reduce with increasing surgeon experience [27]. In Mendivil’s study of participants with a median BMI of 47.9 kg/m^2^, the maximum operative time was lower in the RS group as compared to the open surgery or LS groups, 2.67 h versus 3.92 h and 4.42 h, respectively. Our study shows that rising patient BMI appears to be associated with a small increase in RH operative times, in keeping with other case series [18,24], and is an important consideration in this typically anaesthetically high-risk population when the duration of procedure may influence post-operative outcomes [28]. Increasing surgical team experience with high-BMI cases can streamline the surgical work flow, reducing conversion rates and increasing lymphadenectomy rates [25]. The majority of the patients undergoing gynaecological RH at our institution have a BMI > 40 kg/m^2^ and therefore the lower operating times reported in our study cohort may be a factor of the surgical/anaesthetic team’s experience with this patient population.

The risk of conversion to laparotomy increases with BMI, and studies of RS in patients with BMI > 50 kg/m^2^ report higher conversion rates as compared with lower BMI patients, 19.6% versus 8% [29] and 6.5% versus 4.3% [18], although this is lower than with LS [11]. One of the reasons for this is due to increased intolerance of steep Trendelenburg position and insufflation, which are known to impact on cardiac function [30] and increase the likelihood of complete airway closure [31]. Although BMI was associated with insufflation pressure in our cohort, the use of pressures ≤ 15 mmHg may have helped to increase the tolerability of the procedure, in combination with the lift from the robot arms helping create intra-abdominal space and reducing peak airway pressures, as has been reported previously [32].

The duration of hospital stay was slightly longer than reported in other published case series for RH [24], but comparable to other studies [29]. Non-medical reasons that contributed to hospital stay in our cohort included individuals who lived alone being required to stay for a minimum of two nights and other participants waiting for social services support on discharge. 

The high QOL scores following surgery and short time to self-reported recovery support the use of RH in this population, in keeping with other studies [33,34]. There were a small number of participants who were lost to follow-up at the 12-week time, mainly due to returning to employment. However, all had reported in a previous telephone call that they had returned to previous activity levels. By collecting data at set time points following surgery, we have been able to compare recovery between BMI groups. Previously reported recovery data from patients with high BMI undergoing robotic-assisted surgery have shown that the majority of cases within the obesity category were at the lower BMI range, for example mean BMI 33.7 ± 4.1 kg/m^2^ [35] and in Gaug’s study no participant had a BMI > 50 kg/m^2^ [36]. This highlights the difficulty in extrapolating study results to higher BMI populations and should encourage clinicians to collect data from patients that are representative of the whole surgical population, in order to generate data that can be used in patient counselling and informed consent. Duration of hospital stay and return to previous levels of activity are particularly important in the oncological population, since patients may require adjuvant chemotherapy or radiotherapy, and a longer post-operative recovery could delay treatment, potentially impacting on survival. Apronectomy combined with laparotomy has been reported as an alternative surgical approach for patients with high BMI requiring hysterectomy, enabling greater access to the pelvis [37]. Although this procedure can be associated with longer-term weight loss in a proportion of patients, the duration of surgery is almost one hour longer compared to that for our cohort of patients with a BMI > 50 kg/m^2^, with a mean of 192 min compared to 139.5 min [37]. The length of hospital stay is also longer, with a median of 9 days compared to 2 days in our population. Apronectomy can be associated with a delay in receiving adjuvant therapy, and 24% experienced delays of 3–4 months in receiving adjuvant therapy due to delayed wound healing. The differences in post-operative recovery between the two procedures, the likelihood of long-term sustained weight loss and need for adjuvant therapy should be discussed with patients and assessed on an individual basis. 

### Strengths and Limitations

The major strength of this study is that it was prospective and captured the participants’ self-reported recovery in depth, although a limitation is that this is a relatively small cohort from a single surgical centre. The study aimed to avoid recruitment bias by offering participation to every patient who met the inclusion criteria. However, external validation by data from other surgical centres would enable confirmation of this. Due to the sample size of the study, some results reported in this article have wide confidence intervals due to poor precision. The analyses do not adjust for potential confounders and were limited to univariable associations, as further adjustment could potentially have resulted in misleading or unreliable results. There are some limitations in data collection, including that information on grade ≤ 2 complications was not complete, due to being unable to access primary care records, for example, several participants, when they experienced a small amount of vaginal bleeding or dysuria, were treated empirically with antibiotics, but no infection was demonstrated on microbiological culture. Our study did not contain an objective measure of post-operative recovery, for example, the 6-minute walk test as was used in the ROBOQOL study [38]. We chose instead to use self-reported return to previous activity levels, since this avoided additional hospital visits for assessment and enabled patients to reflect on their personal circumstances and challenges. The post-operative telephone interviews were conducted with a researcher, not the participants’ surgeon, with the aim of encouraging free reporting of symptoms and recovery.

## 5. Conclusions

Patient-reported recovery and quality of life following RH is high in individuals with a high BMI (over 35 kg/m^2^) and does not appear to be impacted by the severity of obesity. 

## Figures and Tables

**Table 1:** Robotic-assisted hysterectomy participant characteristics and body mass index (*N* = 50).

	Total *N* = 50	BMI ≤ 40 *N* = 17	40 ≤ BMI < 45 *N* = 15	45 ≤ BMI < 50 *N* = 8	50 < BMI *N* = 10
BMI	42 [35, 60]	17 (34%)	15 (30%)	8 (16%)	10 (20%)
Age in years	63	63	68	57.5	59.5
	[36, 79]	[40, 77]	[37, 79]	[42, 78]	[36, 71]
ASA classification					
2	20 (40%)	12 (71%)	5 (33%)	3 (38%)	0 (0%)
3	29 (58%)	5 (29%)	10 (67%)	5 (63%)	9 (90%)
4	1 (2%)	0 (0%)	0 (0%)	0 (0%)	1 (10%)
Waist:hip ratio	0.95	0.94	0.94	0.99	0.94
	[0.76, 1.41]	[0.79, 1.02]	[0.81, 1.08]	[0.84, 1.41]	[0.76, 1.07]
Waist	128.5	122	127	141.5	147
	[108, 172]	[108, 137]	[110, 148]	[127, 172]	[117, 170]
Hip	139	131	139	142	155.5
	[117, 182]	[117, 141]	[121, 149]	[122, 158]	[146, 182]
Previous surgery	27 (64%)	10 (59%)	7 (47%)	5 (63%)	5 (50%)
Laparoscopic	9 (21%)	5 (29%)	2 (13%)	1 (13%)	1 (10%)
Open (midline/other)	12 (29%)	2 (12%)	4 (27%)	2 (25%)	4 (40%)
Open (transverse)	6 (14%)	3 (18%)	1 (7%)	2 (25%)	0 (0%)
Histological diagnosis					
CAH	17 (34%)	6 (35%)	4 (27%)	4 (50%)	3 (30%)
CIN/SCC	2 (4%)	0 (0%)	1 (7%)	1 (13%)	0 (0%)
EC	31 (62%)	11 (65%)	10 (67%)	3 (28%)	7 (70%)

The values reported are frequencies (column percentages %) for categorical variables and median [minimum, maximum] for continuous variables. Abbreviations: BMI (Body Mass Index), ASA (American Society of Anaesthesiologists), CAH (Complex Atypical Hyperplasia), CIN (cervical intraepithelial neoplasia), SCC (squamous cell carcinoma), EC (endometrial cancer).

**Table 2:** Intra-operative outcomes for robotic-assisted hysterectomy cases (*N* = 50).

	Total *N* = 50	BMI ≤ 40 *N* = 17	40 ≤ BMI < 45 *N* = 15	45 ≤ BMI < 50 *N* = 8	50 < BMI *N* = 10	Unadjusted Change per Unit Increase in BMI ^1^ (95% CI)	Univariable *p*-Value
Anaesthetic time (mins) ^2^	40.8	38.5	37.7	40.6	48.9	36.6 s	0.018
Mean (SD)	(12.3)	(9.5)	(10.9)	(12.1)	(16.3)	(6.6, 66.6)	
Operative time (mins)	128.1	117.9	129.6	139.4	134.4	65.4 s	0.040
Mean (SD) ^2^	(25.3)	(19.2)	(22.7)	(38.4)	(22.2)	(3.0, 127.8)	
Insufflation pressure (mmHg)	8	8	8	11	12	0.16 mmHg	<0.001
Median [min, max]	[8, 15]	[8, 12]	[8, 15]	[8, 12]	[8, 15]	(0.08, 0.25)	
Length of stay (days)	2	2	2	2	2	0.02 days	0.623
Median [min, max]	[1, 14]	[1, 3]	[1, 14]	[2, 5]	[1, 4]	(−0.06, 0.10)	
Self-reported return to previous activity (weeks) ^3^	4	4	4	6	5	0.03 weeks	0.610
Median [min, max]	[2, 12]	[2, 6]	[2, 12]	[2, 6]	[2, 12]	(−0.09, 0.15)	

^1^ Estimated regression coefficients from univariable linear regression of outcome with BMI included as continuous measure. ^2^ Results exclude 2 (4%) cases who had received additional pelvic lymphadenectomy during RH (*N* = 48). ^3^ Results exclude 2 (4%) cases who had received additional pelvic lymphadenectomy during RH and 2 (4%) participants with missing or vague data for self-reported return to normal activity (*N* = 46). Abbreviations: BMI (Body Mass Index), CI (Confidence interval), SD (Standard Deviation), mmHg (millimetres of mercury).

## Data Availability

Data are unavailable due to ethical restrictions.

## Supplementary Materials

The following supporting information can be downloaded at: https://www.mdpi.com/article/10.3390/cancers15174335/s1, Table S1: Inclusion and exclusion criteria.
