# Supplementary material for: Impact of Patient Body Mass Index on Post-Operative Recovery from Robotic-Assisted Hysterectomy
Source: Cancers (Basel). 2023 Aug 30;15(17):4335. doi: 10.3390/cancers15174335 (PMC10487232; doi:10.3390/cancers15174335)
Supplement: Supplementary file 1 [file cancers-15-04335-s001.zip › cancers-2473665-supplementary.pdf]

Table S1. Inclusion and exclusion criteria.

Inclusion criteria	Women who have been discussed by the gynaecology oncology multi-disciplinary team and then the clinical team have identified them as suitable for robot-assisted hysterectomy ± salpingo-oophorectomy<br><br>Females Aged 18-99 years.<br><br>Participants willing and able to give informed consent for participation in the study.<br><br>No co-morbidities which responsible clinician feels would prevent participant completing the research measures.<br><br>Surgery to be performed by a surgeon experienced in the intended technique.<br><br>Patient BMI ≥ 35kg/m^2
Exclusion criteria	Patients who decline, unable or lack capacity to give consent.<br><br>Where additional operative procedures to those discussed in the inclusion criteria are planned.<br><br>Patients not suitable for minimally invasive surgical management.
